# Ethnomycology of the Guajajara indigenous peoples of Maranhão, Northeast Brazil

**DOI:** 10.1186/s13002-026-00940-2

**Published:** 2026-08-01

**Authors:** Janete Barros da Silva, Ronildo Fernandes Guajajara, Larissa Trierveiler-Pereira, Francisco das Chagas Silva Barros, Roseli Farias Melo de Barros

**Affiliations:** 1 Doutorado em Desenvolvimento e Meio Ambiente, Associação Plena em Rede (PRODEMA/TROPEN), da Universidade Federal do Piauí (UFPI), Teresina, Piauí Brasil; 2 Guia indígena da Aldeia Três Irmãos, Graduando em Licenciatura Intercultural Indígena, pela Universidade Federal do Maranhão, Campus de Barra do Corda, Maranhão, Brasil; 3 Laboratório de Estudos Micológicos, Centro de Ciências da Natureza, Universidade Federal de São Carlos, Campus Lagoa do Sino, Buri, São Paulo, Brasil; 4 Graduando em Licenciatura em Letras Inglês pela Universidade Estadual do Piauí (UESPI), UAB/NEAD, Palmeirais, Piauí Brasil; 5 Docente do Programa de Doutorado em Desenvolvimento e Meio Ambiente, Associação Plena em Rede (PRODEMA/TROPEN), da Universidade Federal do Piauí (UFPI), Teresina, Piauí Brasil

**Keywords:** Mycology, Biodiversity, Ethnomycological knowledge, Uses, Perception

## Abstract

The Brazilian Cerrado harbors a rich diversity of traditional communities, including several Indigenous peoples. Among them are the Guajajara, who inhabit regions extending from the state of Maranhão to the northeastern portion of Pará. Considering the importance of research into ethnomycological knowledge in indigenous communities for the preservation of culture and the environment, especially in relation to macrofungi, the aim of this study was to document and compile the uses of macrofungi by the original Guajajara peoples to evaluate and revitalize traditional knowledge among indigenous generations. The study was carried out in the Cana Brava reserve, located in Barra do Corda, in the state of Maranhão (northeast Brazil). The methodological approach used rapport techniques, semistructured interviews, guided tours, checklist interviews with photo albums and fresh or dried fungal samples, field notebooks and quantitative analysis via Use Value. Ninety-six indigenous people from the Pé de Galinha (28.2%) and Três Irmãos (71.8%) villages were interviewed, 54.1% female and 45.9% male. Participants were recruited through purposive sampling based on the number of families residing in the community who were willing to participate in the study, after obtaining authorization from community leaders. Discourse analysis revealed that Guajajara once used at least six of the 42 species documented. Although they no longer use them in their diet, they agree that *Auricularia tremellosa* (Fr.) Pat. was used in the diet of their ancestors. For recreational purposes, they use *Calvatia rugosa* (Berk. & M.A. Curtis) D.A. Reid, *Geastrum hariotii* Lloyd. and *G. javanicum* Lev. and occasionally for medicinal purposes: *Pycnoporus sanguineus* (L.) Murrill and *Lentinus crinitus* (L.) Fr. The study communities can be considered partially mycophilic. Vertical transmission is responsible for the dynamics of macrofungal knowledge. This study can contribute both to the appreciation and conservation of traditions and to Brazilian fungal biodiversity.

## Introduction

Ethnomycology is an area of research focused on the interaction of fungi with local human communities [1–3]. Owing to their ecological, medicinal, nutritional and health-promoting properties, macrofungi are gaining interest among scientific and research communities around the world [4]. They have macroscopic reproductive structures, called ascomata and basidiomata, and are important representatives of Ascomycota and Basidiomycota [5].

Various estimates have been proposed to elucidate the number of fungal species worldwide [6–8]. The most widely accepted estimates between 2.5 and 3 million species were proposed and revised by [6, 7]. The few ethnomycological studies carried out in Brazil have mostly focused on indigenous populations in the Amazon [9, 10].

In addition, ethnomycological studies on indigenous ethnic groups in Brazil are relevant because of the research of the British Botanist Ghillean Prance in 1967 with indigenous Yanomamis from the Amazon. In the 1970s, Prance discovered the use of mushrooms as part of the Yanomami diet [11, 12]. In addition, on the ethnomycology of Caiabi, Txicão and Txucurramãe brought valuable contributions between the original peoples and the consumption of macrofungi [13].

Indigenous communities have traditionally applied their traditional knowledge to the collection of wild macrofungi for a variety of purposes, including medicinal, recreational and dietary uses. In many contexts, these fungi have been considered secondary food resources [14, 15].

Ethnomycological research in Brazil has gone through two periods: utilitarian and cognitivist [16, 17]. The utilitarian ones, carried out by naturalists and anthropologists, took place before the 1960s and described the use of fungi by different Brazilian indigenous groups in a generic way. On the other hand, research with a cognitive bias was marked by the work of [18].

Ethnomycological research in Brazil is currently experiencing what has been described as a third phase [19], referred to as the period of diversity. In this stage, research has expanded beyond the ethnomycological knowledge of Amazonian Indigenous peoples to include other social groups and communities, such as traditional populations (e.g., quilombola communities) as well as local and rural communities across different regions of the country [20–26].

This recent phase has also broadened the scope of ethnomycological investigations by incorporating new research approaches and contexts, including perception studies and educational initiatives related to mycology [27]. Such studies have been conducted in rural and indigenous schools in Brazil, contributing to a better understanding of how knowledge about fungi is perceived, transmitted, and integrated within different cultural and educational settings [28, 29].

Despite the growing body of ethnomycological research in Brazil, studies focusing on traditional and Indigenous communities remain limited in several regions of the country. Documenting and understanding local knowledge systems is essential for preserving biocultural diversity and for advancing ethnomycological research globally. In this context, the local knowledge of the Guajajara people from Maranhão represents an important opportunity to investigate how macrofungi are perceived, used, and culturally transmitted within Indigenous societies.

Therefore, this study addresses the following research questions: How are macrofungi perceived within the local environment, and how is ethnomycological knowledge transmitted across generations? Additionally, which macrofungal species are recognized and used by the Guajajara people in Barra do Corda, Maranhão, Brazil?

This study aimed to document and compile the uses of macrofungi by the indigenous Guajajara people who live in the Cana Brava reserve, Barra do Corda-Maranhão, to evaluate and recover ethnomycological knowledge.

## Materials and methods

### Study area

**Fig. 1:**
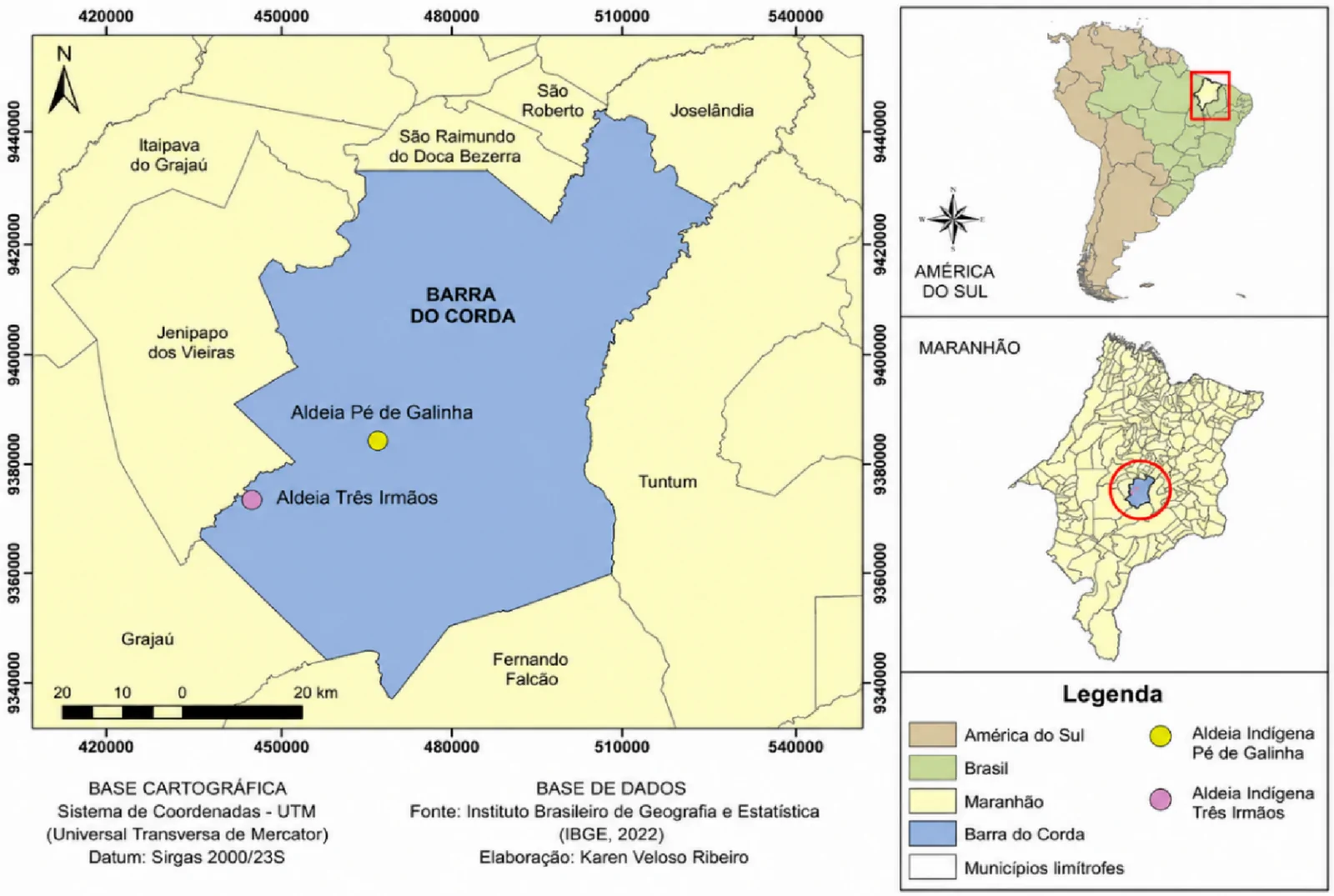
Location of the Pé de Galinha and Três Irmãos Indigenous Villages in the Cana Brava Reserve, municipality of Barra do Corda, Maranhão, Brazil

The villages of Pé de Galinha (5^°^29’ 30” S; 45^°^14’23” W) and Três Irmãos (5° 40’00” S; 45^°^29’43.0” W) are located in the Cana Brava Indigenous Reserve near the city of Barra do Corda in Maranhão (Northeast Region of Brazil) and are 45 km from the city’s urban center (Fig. 1). The municipality is located in the Alto Mearim and Grajaú microregion, 450 km from the capital of Maranhão, São Luís, and 350 km from the capital of Piauí, Teresina [30].

Barra do Corda has a population of 84,532 inhabitants and a per capita of 10,004.24. It has a land area of 5,187.673 km² and a population density of 16.29 inhabitants per km² in the Cerrado region [30]. In Maranhão, the climatic seasons are well defined, with a rainy season from January to May, a dry season from June to December and a semihumid climate [31].

With very diverse vegetation, the state has the formations of the Amazon Rainforest in the west, the Cocais Forest in the east, mangroves in the coastal region and the Cerrado. The main indigenous lands include Alto Turiaçú, Araribóia, Carú, Awá, Krikati, Cana Brava, Kanela, Bacurizinho and Porquinhos, lands that are home to the Guajajara and Canela do Maranhão ethnic groups [32].

According to Coelho (1987), the villages of Guajajara extend from the municipality of Barra do Corda/MA to the northeastern state of Pará. Those located in Pará are called Tembé but share the same language and cultural traditions [33]. Guajajara also use Portuguese to communicate.

### Ethical aspects of the research

The ethnomycological study, which was both qualitative and quantitative, was approved by the Human Research Ethics Committee (CEP) of the Federal University of Piauí (UFPI), with opinion number 6.737.491, by the National Research Ethics Commission (CONEP), with number 6.971.260, The National Foundation for Indigenous Peoples (FUNAI), under number 000173.0012899/2023, the National System for the Management of Genetic Heritage and Associated Traditional Knowledge (SISGEN), under number A360372 and the SISBIO, under number 84,798.

Before each interview, the informant signed the informed consent form, in compliance with Resolutions 304/2000 and 510/2016 of the National Health Council (CNS), to clarify possible doubts about the work, risks and/or benefits, and possible damage that may arise in the research, in addition to the Consent for Use of Image. Up to three people from the same household, a couple, daughters or sons of legal age (> 18 years), were interviewed individually to verify the transmission of knowledge.

### Study sample

Pé de Galinha village comprises 12 families (58 indigenous people), whereas Três Irmãos village comprises 52 families (approximately 230 indigenous people), including individuals ranging from infants to elderly. Participants were recruited through purposive sampling based on the number of families residing in the community who were willing to participate in the study, after obtaining authorization from community leaders. The sample size was determined using the Comentto sample size calculator, adopting a 95% confidence level and a 5% margin of error. A total of 96 Guajajara participants, including men and women, were interviewed: 27 individuals from Pé de Galinha village (28.2%) and 69 from Três Irmãos village (71.8%). Discourse analysis indicated that the Guajajara people have historically used at least six of the 42 macrofungal species documented in this study.

The interviewees were classified according to the age group [30] as young people (18 to 24), adults (25 to 59) and elderly individuals (aged 60 and over). Among those interviewed, 20 were young people, 52 were adults, and 24 were elderly. The sample universe was established via the Comentto calculator (https://comentto.com/calculadora-amostral/), with a 95% confidence level and a 5% margin of error [34].

### Techniques and field research

To build rapprochement and trust, the *rapport* technique was used, which, is necessary to strengthen contact with indigenous people and to ensure that the researcher is allowed to stay in the field [35]. A pilot excursion in September 2021 established the first contact with indigenous people, which was mediated by a religious leader (Matusalém Braga), to obtain the chiefs’ consent to carry out the study. A total of 15 field trips were conducted, each lasting three to five days, with the objective of collecting data. These excursions took place during the months of January to May, the rainy season, and were best suited for collecting macrofungi. Semi-structured interviews were then conducted from August to November.

**Fig. 2:**
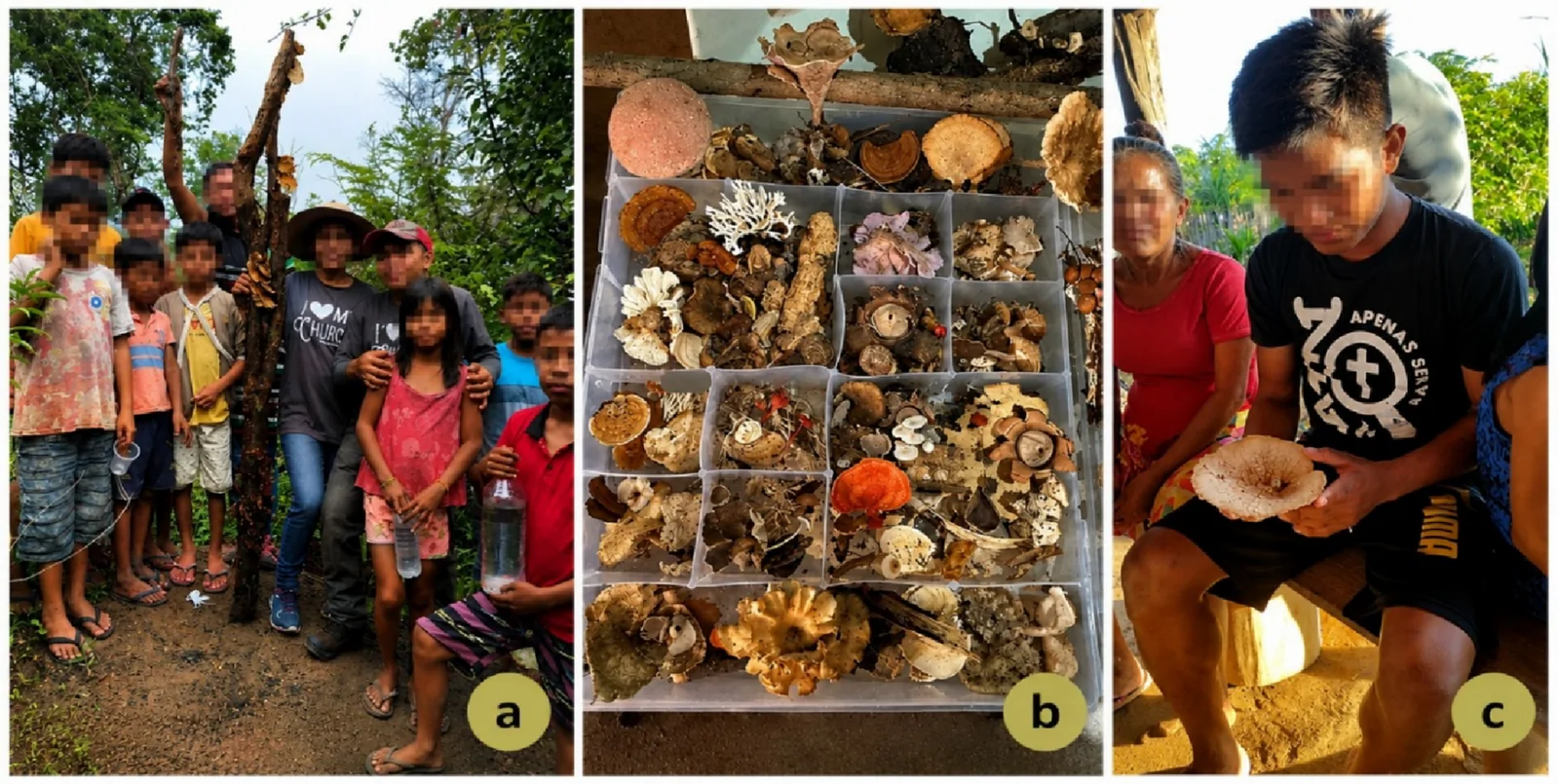
Collection of macrofungi in the Cana Brava Indigenous Reserve, Barra do Corda/MA. (**a**) Collection team from Pé de Galinha village. Source: Ronildo Guajajara. (**b**) Macrofungi for sampling at the time of the interview; (**c**) Indigenous person from Pé de Galinha village observing a macrofungus. Source: Janete Barros da Silva

The semi-structured interviews [36] were carried out via forms with open and closed questions about socioeconomic data (gender, age, schooling, profession, length of time living in the community, monthly income, and type of housing) and information about fungal knowledge and its uses, food, medicine, and recreation [37]. At the same time, the Checklist interview technique [35] was used, which uses a photographic catalog as a visual stimulus to recover ethnomycological information [38] about the species occurring in the study area (Fig. 2).

For this purpose, photographs were taken of the macrofungi occurring around the village and nearby forests, in addition to collecting the samples. These photographs were printed in color on an Epson (375) multifunction printer, A4 sheet size (297 × 210 millimeters to be used during the interviews. In addition to the images, macrofungal samples (fresh or dried) were used.

### Collecting ethnomycological data

Mycological material and vernacular names was collected via guided tours [35, 39]. Collections were carried out on different substrates (trunks, thin branches, soil and litter or leaf litter) [40]. At the time of collection, the macrofungi were measured and photographed (RedMi 9pro camera/cell phone and iPhone 15), and their morphological characteristics (size, color, substrate) were documented [41].

The samples were packed in transparent collection boxes, separated so as not to damage the structures and then transported in fresh cardboard boxes to observe their structures for macro- and microscopic identification. To help with identification, the study relied on the partnership of LEMic-UFSCar (Laboratory of Mycological Studies at the Federal University of São Carlos), coordinated by taxonomist Larissa Trierveiler Pereira, who was responsible for the identification of the fungal species [10, 15, 42, 43]. The samples were then herborized and deposited in the collection of the Graziela Barroso Herbarium (TEPB) at the Federal University of Piauí (UFPI).

To investigate knowledge about folk fungal taxonomy, participants were asked the following questions: “What is this and how do you know it?” and “Which ones do you use (for food, medicine, or recreation)?” The aim was to obtain data on macrofungi, including vernacular names, uses, notions of folk taxonomy, ecological aspects, and the transmission of knowledge through local narratives.

Furthermore, the modes of transmission of ethnomycological knowledge within the community were identified, occurring through three main pathways: [4] vertical or intragenerational transmission, carried out among relatives, usually between parents and children; [39] horizontal transmission, which occurs among individuals of the same generation, regardless of kinship ties; and [44] oblique transmission, mediated by external sources of information such as digital technologies, the internet, books, television, and radio [45].

### Data analysis

The Mycobank database (www.mycobank.org/name) was used to look up the spelling of species and family names, as well as the abbreviations of authors’ names [46]. We used the Use Value (UV) [47–49], where the sum of the number of uses given by the informant (U) is divided by the total number of informants (n) who cited it, thus obtaining the following formula: UV=(ΣU)/n. In the analysis, a distinction was made between citations of current use value (current VU) and potential use value (potential VU).

This index therefore assumes that the relative importance of a macrofungus is indicated by the number of uses it has. Current Use Value (VU) represents the present-day uses of macrofungi reported by the informants, while potential (VU) includes historical or previously reported uses that are no longer practiced by the community [47, 50].

### Return to the community

A workshop was held at the community school, with the participation of children, young people and adults. On that occasion, various visual resources were used to obtain data, including books, printed photographs of the macrofungi found in the village, fresh and dried samples arranged in compartmentalized boxes, as well as audio and video materials.

## Results

### Profile of the interviewees

The research participants presented different levels of education: 27% were illiterate, and in these cases, consent was recorded through fingerprint signatures on the Free and Informed Consent Form. In addition, 42% had incomplete elementary education, 29% had incomplete secondary education, and only 2% had higher education. It was observed that all interviewees had lived in the villages for more than 14 years, indicating a strong connection with the territory and local cultural practices.

Regarding occupational activities, women were involved in domestic tasks, agricultural activities, and handicraft production, while men mainly worked in fishing, hunting, and agriculture. The main sources of subsistence included the cultivation of cassava, manioc, corn, rice, and sweet potatoes, in addition to animal husbandry and hunting and fishing practices. Handicraft production, such as hammocks, necklaces, bracelets, feather headdresses, maracas, and wooden toys, also stood out as a relevant activity, both culturally and economically. Additionally, part of the families received support from governmental social programs.

### Recognition and diversity of macrofungi

**Fig. 3:**
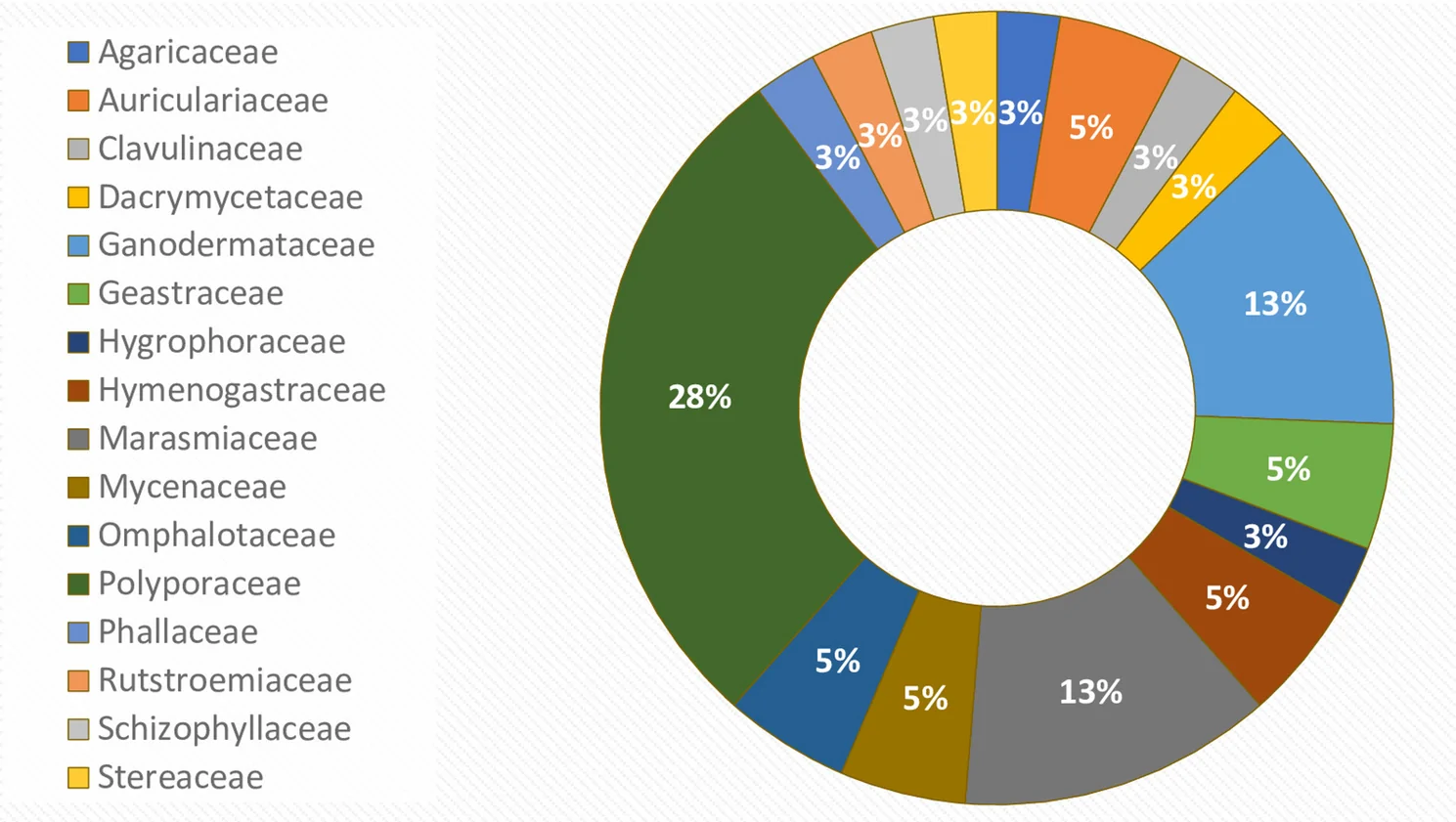
Representativeness of species the families of the Phyla Basidiomycota and Ascomycota identified in the Três Irmãos and Pé de Galinha Villages, Cana Brava Indigenous Reserve, Barra do Corda - MA, Brazil.

Macrofungi belonging to 16 families were recorded, distributed within the phyla Basidiomycota (15 families) and Ascomycota (one family: Rutstroemiaceae). The greatest diversity was observed in the phylum Basidiomycota, which was expected due to the predominance of macroscopic fungi in this group (Fig. 3).

Among the identified families, Polyporaceae showed the highest species richness, standing out in comparison to the others. Ganodermataceae and Marasmiaceae were also relevant, although with fewer species when compared to Polyporaceae.

In the ethnobiological context, it was observed that the term “urupé,” of Tupi-Guarani origin, is widely used to designate macrofungi and is employed by the Guajajara people for different species. The attribution of local names proved to be limited and was frequently associated with the environment of occurrence (such as the forest) or with similarities to plants, indicating a classification system based on general and perceptual characteristics.

### Intergenerational transmission of ethnomycological knowledge

The transmission of ethnomycological knowledge among the Guajajara occurred predominantly through vertical, or intrafamilial, transmission, representing 62% of the reported cases. This result highlights the importance of the family as the main nucleus for the conservation and dissemination of traditional knowledge in the villages studied.

Regarding transmission agents, men were more frequently mentioned (71%) compared to women (29%). This pattern may be related to activities traditionally performed by men, such as hunting, fishing, and agriculture, which favor greater interaction with the forest environment and, consequently, with macrofungi.

Qualitative reports reinforced these quantitative data, indicating that knowledge about macrofungi is also associated with healthcare practices and traditional medicine. Some interviewees highlighted the use of these organisms in the treatment of illnesses, especially by local healers. One participant reported personal experiences involving the medicinal use of macrofungi, demonstrating the relevance of these resources within the context of traditional healthcare.

### Macrofungi species and their potential uses

Among the documented species, six were cited regarding their potential uses, distributed into the medicinal, edible, and recreational categories. The species *Pycnoporus sanguineus* and *Lentinus crinitus* were associated with medicinal use, being mainly indicated for the treatment of gastrointestinal disorders and other conditions reported by the participants.

The species *Auricularia tremellosa* was mentioned as edible, with reports indicating its traditional consumption during forest activities, such as hunting, especially during colder periods.

Regarding recreational use, the species *Calvatia rugosa*, *Geastrum javanicum*, and *Geastrum harriotii* were cited as being used by children and young people in playful activities. This type of use has also been described in the literature for rural communities, especially involving fungi with structures that release spores when handled, which arouses children’s interest and interaction.

Overall, the data indicate that although the number of species used is relatively limited, macrofungi play multiple roles in the daily life of the community, encompassing medicinal, nutritional, and cultural aspects.

### Frequency of use and cited species

The use value was calculated for six macrofungi species, totaling 24 citations (Table 1). Polyporaceae was the most cited family, followed by Marasmiaceae. In the recreational category, Calvatia rugosa had the highest number of mentions (*n* = 7), mainly among younger participants.

**Table 1:** Occurrence and diversity of macrofungal species documented in the Pé de Galinha and Três Irmãos villages, Cana Brava Indigenous Reserve, Barra do Corda - MA, Brazil

Family/species	SUB	USES	CN	CUV	PUV
1. Agaricaceae Chevall. *Calvatia rugosa* (Berk. & M.A. Curtis) D.A. Reid *Leucocoprinus birnbaumii* (Corda.) Cantora *Leucocoprinus cretaceus* (Bull.) Locq.	SOL DTR DTR	REC NOT NOT	7 0 0	0.20 - -	0.08 - -
2. Auriculariaceae Fr. Ex Lindau *Auricularia cornea* Ehrenb. *Auricularia tremellosa* (Fr.) Pat.	DTR DTR	NOT COM	0 2	- 0	0 0.08
3. Clavulinaceae Donk *Clavulina cristata* (Holmsk.) J. Schröt.	SOL	NOT	0	-	-
4. Dacrymycetaceae Bref. *Dacryopinax spathularia* (Schwein.) G.W. Martin	DTR	NOT	0	-	-
5. Ganodermataceae (Donk) Donk *Amauroderma exile* (Berk.) Torrend *Amauroderma rude* (Berk.) Torrend *Amauroderma* sp. *Ganoderma lucidum* (Curtis) P. Karst. complex *Ganorderma* sp.	LTR LTR LTR DTR DTR	NOT NOT NOT NOT NOT	0 0 0 0 0	- - - - -	- - - - -
6. Meruliaceae P. Karst. *Flavodon flavus* (Klotzsch) Ryvarden	DTR	NOT	0	-	-
7. Geastraceae Corda *Geastrum hariotii* Lloyd. *Geastrum javanicum* Lév.	SOL SOL	REC REC	4 5	0.12 0.12	0.08 0.04
8. Hygrophoraceae Lotsy *Hygrocybe coccinea* (Schaeff.) P. Kumm.	SOL	NOT	0	-	-
9. Hymenogastraceae Vittad. *Pleurotus ostreatus* (Jacq.) P. Kumm. *Phellinus gilvus* (Schwein.) Pat. *Psilocybe cubensis* (Earle) Singer *10. Marasmiaceae Roze ex KühnerMarasmiellus ramealis* (Touro) Cantor *Marasmiellus* sp. *Marasmius sullivantii* Mont. *Marasmius* sp.1 *Marasmius* sp.2 *Trogia cantharelloides* (Mont.) Pat.	DTR DTR DUN MLE DVN DTR MLE MLE SOL	NOT NOT NOT NOT NOT NOT NOT NOT NOT	0 0 0 0 0 0 0 0 0	- - - - - - - - -	- - - - - - - - -
11. Omphalotaceae Bresinsky *Gymnopus* sp.1 *Gymnopus* sp.2	SOL SOL	NOT NOT	0 0	- -	- -
12. Polyporaceae Corda *Hexagonia hydnoides* (Sw.) M. Fidalgo *Lentinus brumalis* (Pers.) Zmitr. *Lentinus crinitus* (L.) Fr. *Fomes fomentarius* (L.) Fr. *Pycnoporus sanguineus* (L.) Murril *Trametes betulina* (L.) Pilát *Trametes versicolor* (L.) Lloyd *Trametes* sp. *Polyporus* sp.	LTR SOL SOL SOL LTR DTR DTR DTR DTR	NOT NOT MED NOT MED NOT NOT NOT NOT	0 0 0 0 0 0 0 0 0	- - - - - - - - -	- - - - - - - - -
13. Phallaceae Corda *Phallus indusiatus* Vent.	SOL	NOT	0	-	-
14. Rutstroemiaceae Holst-Jensen, L.M. Kohn & T. Schumach. *Rutstroemia echinophila* (Bull.) Höhn.	DTR	NOT	0	-	-
15. Schizophyllaceae Quél. *Schizophyllum commune* Fr.	LTR	NOT	0	-	-
16. Stereaceae Pilát *Stereum ostrea* (Blume & T. Nees) Fr.	DTR	NOT	0	-	-

Janete Barros da Silva, 2025

Substrate (SUB): Soil (SOL); Decomposing trunk (DTR); Living trunk (LTR); Membranaceous leaf (MLE); Dung (DUN); Not cited (NOT). Use value: No use value (–). Citation number (CN); Current use value (CUV); Potential use value (PUV). Uses: Recreational (REC); COM (edible); MED (medicinal)

The species *P. sanguineus* was cited five times for medicinal use, especially in the treatment of conditions related to the female genital tract, kidneys, and back pain, being used in the form of a decoction. The reported instructions included preparing the tea by boiling the macrofungus in water, with daily consumption until symptom improvement.

### Medicinal uses and body systems

The medicinal uses were classified into four body systems according to categories established by the World Health Organization. *P. sanguineus* presented the highest use value, with applications associated with the female reproductive system, renal system, and musculoskeletal system. *L. crinitus*, in turn, was related to the treatment of gastrointestinal system disorders.

Qualitative reports reinforced these data, including descriptions of the traditional use of *P. sanguineus* in specific situations, such as the control of postpartum hemorrhage, indicating the cultural and therapeutic relevance of this species in the past.

### Usage trends and cultural changes

In general, a low current use value of the species was observed among the Guajajara, suggesting a partially mycophilic relationship. This result may be associated with the reduction in the traditional use of macrofungi, possibly replaced by access to pharmaceutical medicines. The case of *A. tremellosa* illustrates this change, since although it was consumed in the past, there is currently no recorded use among the interviewees.

The use value index reflects the cultural importance attributed to species based on citations, not necessarily indicating their current use. In this context, it was observed that species belonging to the orders Geastrales and Polyporales concentrated the highest number of mentions, encompassing different categories of use and body systems.

### Context of macrofungi collection

The macrofungi used by the Guajajara are traditionally collected from the natural environment, especially during activities in the forest or from decomposing logs. No cultivation practices were observed, reinforcing the dependence on local ecological knowledge for their acquisition.

## Discussion

### Recognition and use of macrofungi

In the indigenous communities studied, the recognition and use of macrofungi proved to be limited, with few species cited as being used by the Guajajara indigenous people, indicating a low degree of familiarity with and utilization of these organisms in the local context. This result suggests a reduced incorporation of macrofungi into the Guajajara’s dietary and cultural practices, differing from other ethnomycological contexts in which these resources play a more significant role.

However, it is observed that the selection of the mentioned species does not occur randomly, but is associated with limited medicinal practices, such as indications for the treatment of diseases of the female reproductive system, as well as recreational or playful activities. This pattern of restricted and selective use has been described in the literature as reflecting both cultural preferences and processes of loss or discontinuity of traditional knowledge [14, 68].

In this study, the diversity of the phylum Basidiomycota was greater than that of the phylum Ascomycota, which was expected, considering that most macroscopic fungi belong to the basidiomycetes, a group that includes several widely known representatives, such as edible mushrooms [22].

Within Basidiomycota, the family Polyporaceae stood out for presenting the greatest species richness, with a total of 42 documented species (Table 1). This result is consistent with previous studies that identify Polyporaceae as one of the most diverse fungal families in Brazil [51].

Representatives of this family are characterized by generally stipitate basidiomata and a dimitic hyphal system, composed of skeletal-binding hyphae or of the “bovist” type, characterized by a central axis with arboriform branches, frequently dichotomous, ending in thin extremities. In addition, they possess generative hyphae with clamp connections and cylindrical to subcylindrical basidiospores [10].

In the ethnomycological context, it is observed that in the Tupi–Guarani language, the term used to designate mushrooms is *urupê*. Similarly, the Guajajara people employ the term *urupé* to refer to these organisms. Although similarly spelled, these terms present phonetic variations in Portuguese, reflecting particularities of local linguistic use [52].

In the studied villages, a low diversity of names attributed to macrofungi was recorded (Table 1), these being generally associated with the environment of occurrence, such as the “forest,” or with similarities to “plants.” The term *urupé*, for example, may be used to designate different species. This pattern corroborates previous studies and indicates that, in some indigenous cultures, fungi are frequently recognized and classified in ways similar to plants [49].

In contrast, the Caiabi people from northern Brazilian Amazonia use the term *uepó*, mainly for fungi that grow on trees (with one exception), and add adjectives to distinguish different types [9]. The Sanoma subgroup of the Yanomami use the prefixes *parólito* or *uonchêlá*, with different combinations of specific adjectives, to distinguish between edible and non-edible fungi belonging to the families Auriculariaceae, Clavariaceae, Polyporaceae, Strophariaceae, and Xylariaceae [53].

Indigenous populations in Brazil present two distinct patterns of fungal classification. Some ethnic groups possess a very simple vocabulary and name fungi only according to a property or resemblance that may characterize them, whereas others demonstrate a broader perception of different fungi, evidenced by a complex vocabulary in which classificatory lexemes are used to refer to different fungal taxa [9].

### Transmission of knowledge

In this study, vertical or intrafamilial transmission (62%) was the main process possibly responsible for the spatial dynamics of local knowledge about macrofungi in the investigated villages. The data indicate that the transmission of ethnomycological knowledge presents a gender dimension, with male predominance (71%), possibly associated with hunting, fishing, and agricultural activities, which favor greater interaction with forest environments, as observed in southeastern Poland by [54]. According to the literature, gender was one of the main variables influencing the distribution of local knowledge [55].

In Geneva, Switzerland, mushroom gathering is predominantly carried out by men, who also possess greater mycological knowledge and transmit it more frequently across generations. This differs from Mexico, where women participate in the entire process of collecting, selling, handling, learning about ecology and taxonomy, as well as teaching about macrofungi [56]. In rural communities of northeastern Piauí, women also play a leading role in the collection and dissemination of information about fungi [25].

The horizontal mode of transmission (17%) was the least mentioned by interviewees, indicating that knowledge exchange among peers does not constitute the main pathway of ethnomycological learning. Even so, it was observed that 21% of participants reported the influence of external agents within the family context, characterizing oblique transmission, with emphasis on the school as a space that positively contributes to the acquisition and expansion of this knowledge [57]. In addition, a small proportion of young people (8%) demonstrated interaction with species through playful activities and games, which may represent a complementary pathway for familiarization with fungi [58].

In this context, the predominance of vertical transmission, based on oral tradition and intrafamilial coexistence, is consistent with studies conducted on medicinal plants and fungi in northeastern Brazil, in which traditional knowledge is mainly shared among family members [26, 59, 60]. These findings reinforce that, although horizontal and oblique contributions exist, the family nucleus remains the main structural axis for the transmission of traditional knowledge.

A recent study evaluated how ethnomycological knowledge is transmitted among people from indigenous communities in Mexico. Elderly individuals in these communities possessed greater knowledge about the traditional use of wild mushrooms and were responsible for transmitting it to younger generations. Men collected and knew the mushrooms that grow in forest areas distant from the villages, whereas women and children collected them in areas closer to their homes [61]. Thus, traditional knowledge regarding the use of macrofungi is orally transmitted from generation to generation [22, 44, 56, 62].

### Uses of macrofungi (medicinal, food, and recreational)

**Fig. 4:**
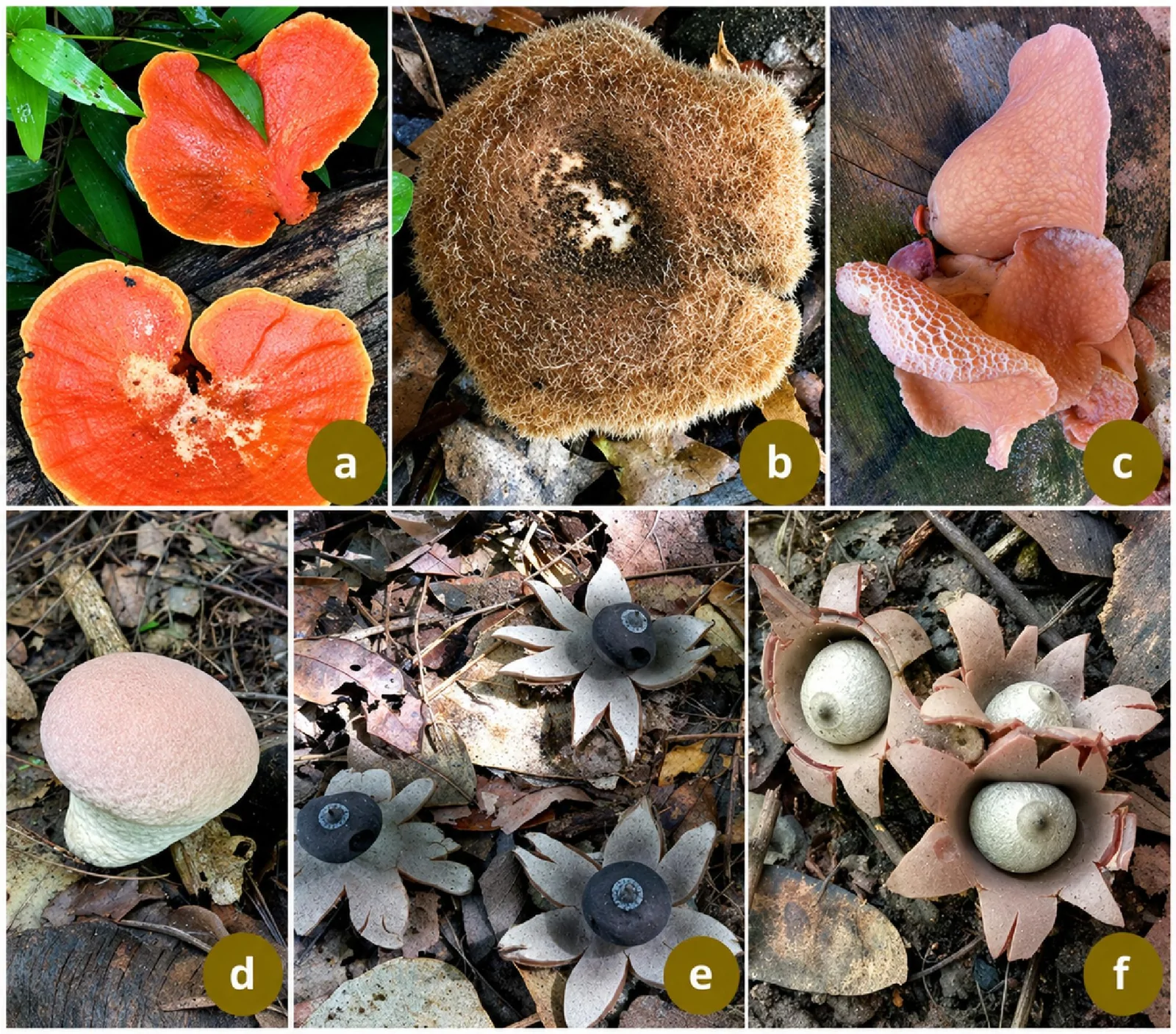
Macrofungal species cited by the Guajajara indigenous peoples of the Três Irmãos and Pé de Galinha villages in the Cana Brava Reserve, Barra do Corda, Maranhão, for medicinal. (**a, b**) edible (**c**) and recreational (**d, e, f**) uses. (**a**) Pycnoporus sanguineus, (**b**) Lentinus crinitus, (**c**) Auricularia tremellosa, (**d**) Calvatia rugosa, (**e**) Geastrum harriotii, (**f**) Geastrum javanicum Source: Janete Barros da Silva

Some methodological limitations of the use of questionnaires should be considered. The fact that interviewees reported recognizing certain mushroom species does not necessarily imply that they know how to use them, and may only indicate familiarity with their names [54]. Even so, among the documented species, six were cited with specific uses, distributed into the medicinal (*P. sanguineus* and *L. crinitus*), recreational (*C. rugosa*, *G. harriotii*, and *G. javanicum*), and edible (*A. tremellosa*) categories (Fig. 4).

Ethnographic reports highlight the historical relevance of the medicinal use of macrofungi. One interviewee reported that, in the past, “urupés” were widely used by older people in the treatment of diseases. He also mentioned the work of a healer who used macrofungi in the form of tea (decoction), including *P. sanguineus*, to treat diseases of the female reproductive tract and kidneys. In his testimony, he stated: “I was almost dying from severe stomach pain; I went to the healer, he gave me the tea to drink, and I was soon cured.” In another case, he reported: “My son’s wife lost a lot of blood while giving birth (…), we went to the healer and he gave her orange urupé to drink as tea, and soon the bleeding stopped” (male, 74 years old).

The use of tea preparations was also recurrent in the reports. Participants indicated that their grandparents medicinally used “wood ear” fungi (Basidiomycota), reinforcing the intergenerational continuity of this traditional knowledge [22].

Figure 4 presents the macrofungi species cited by the Guajajara indigenous people from the Três Irmãos and Pé de Galinha villages, in the Cana Brava Reserve, Barra do Corda, Maranhão, for medicinal, food, and recreational uses, including *P. sanguineus*, *L. crinitus*, *A. tremellosa*, *C. rugosa*, *G. harriotii*, and *G. javanicum*.

The medicinal use of *P. sanguineus* is also widely documented in other ethnobiological contexts. Among the Caiabi ethnic group in northern Brazil, the species was recorded as a treatment for intense nightmares [13]. In rural communities of northeastern Brazil, it is cited as a menstrual cycle regulator [63], in addition to presenting anti-hemorrhagic and anti-inflammatory properties [45]. In different regions of Mexico, indigenous populations use this species for various purposes, including the treatment of inflammations and cosmetic applications [64].

These traditional uses are supported by recent scientific evidence. Biochemical studies demonstrate that *P. sanguineus* possesses anti-inflammatory activity and high therapeutic and pharmacological potential, in addition to applications in the biological control of phytopathogens such as *Botrytis cinerea* and *Fusarium oxysporum* [65]. These findings reinforce the relevance of traditional knowledge as a basis for scientific investigations and possible biotechnological applications.

Another medicinal species mentioned was *L. crinitus*, known as “hairy urupé” by the Guajajara. This species is used in the treatment of gastrointestinal tract diseases. One interviewee (male, 69 years old) reported: “Strong to cure stomach pain, diarrhea (dysentery); in the absence of medicine from the city, we use urupé as tea and get better, and it is found in the forest where we live.” This testimony reinforces the importance of macrofungi as a therapeutic alternative in contexts with limited access to conventional medicine, highlighting the dependence on local natural resources.

Regarding food use, *A. tremellosa* was cited as an edible species. Two interviewees (males, 49 and 61 years old) reported that their parents consumed this macrofungus raw during hunting activities: “our parents used to go hunting in the forest during cold weather, and they found the ‘soft-brown-flower’ urupé on logs and ate it to endure the hunt and then return to the village.” This use demonstrates not only the nutritional value of the species, but also its relevance as an energy resource in traditional activities.

The consumption of *A. tremellosa* is not restricted to the local context, being widely recorded in different regions of the world. Studies on the diversity of the genus *Auricularia* demonstrate its broad global distribution and highlight its edible and medicinal properties, with records across several continents. In addition, there is evidence of its consumption by ancient populations, often being cooked with beans and incorporated into traditional preparations [22].

The food use of macrofungi is also widely documented among indigenous peoples and traditional communities. In the Colombian Amazon, the Uitoto, Muinane, and Andoke peoples use fungi as a food source [66]. Similarly, consumption has been recorded among the Hotï people of the Venezuelan Amazon [33] and among rural and riverside populations of the Peruvian Amazon [67]. Among the Yanomami (Sanuma), fungi are generally boiled before consumption or roasted in banana leaves, although they are described as having a mild flavor [53]. These records demonstrate the diversity of culinary practices associated with macrofungi.

In addition to food and medicinal uses, macrofungi also play cultural and recreational roles. Among the Karajá indigenous people, for example, fungi are mainly used for playful and ornamental purposes during festivities, with occasional use in traditional medicine [49]. Similarly, in the present study, three species were cited: *C. rugosa*, *G. javanicum*, and *G. harriotii*, used by children and young people in games.

This playful use has already been described in other communities, especially involving fungi known as “puffballs,” which release clouds of spores when pressed, attracting children’s interest [22]. In the state of Piauí, species of the genera *Agaricus* and *Coprinus* have also been recorded as play objects in rural communities [45]. In Latin American contexts, similar reports exist in Mexico, where species such as *Cookeina sulcipes* and *Cookeina tricholoma* are used in playful activities [43, 68].

### Use value of macrofungi

The use value (UV) of macrofungi consists of a quantitative index employed to estimate the relative importance of a given species based on the frequency of citations by a group of informants. This indicator allows the inference of the degree of knowledge and interaction between human communities and fungal resources and may vary significantly among different sociocultural contexts. In the present study, a low use value was observed for the recorded species, suggesting only a partially mycophilic relationship, possibly associated with the reduced current use of these organisms.

This pattern contrasts with results reported for traditional communities in the Philippines, where high use values were recorded for several macrofungi species. In that context, *V. volvacea* presented a use value of 0.86 and was considered the most popular edible mushroom in the region. Such findings led to the classification of these communities as mycophilic due to their strong cultural affinity and recurrent use of macrofungi [19].

In the present study, the use value was calculated for six species, totaling 24 citations among the indigenous informants (Table 1). Among the identified families, Polyporaceae was the most representative family in terms of number of citations, followed by Marasmiaceae. In the recreational category, *C. rugosa* stood out with the highest number of mentions (*n* = 7), especially among younger individuals. In turn, *P. sanguineus* was cited five times for therapeutic uses related to the treatment of disorders of the female reproductive tract, kidneys, and lower back pain. The preparation consisted of decocting the basidioma for approximately 30 min in one liter of water, with the infusion being consumed once a day, on an empty stomach, until symptom remission.

Additional ethnographic reports indicated the use of *P. sanguineus* in specific situations, such as the control of postpartum hemorrhage. Informants from the same family nucleus described the use of “orange urupé” in the form of tea, administered shortly after childbirth in a case of severe bleeding, which, according to the report, ceased after ingestion of the tea preparation.

The recorded medicinal uses were classified into four body systems according to categories proposed by the World Health Organization. *P. sanguineus* presented the highest use value, with indications associated with the female reproductive system, urinary system, and musculoskeletal system. *L. crinitus*, in turn, was related to the treatment of gastrointestinal system disorders.

It is important to emphasize that the use value index does not necessarily reflect the current frequency of use of a species, but rather indicates the existence of traditional knowledge associated with its use, whether current or past. In this sense, a greater number of citations was observed for species belonging to the orders Geastrales and Polyporales. In addition, informants reported applications involving multiple body systems, highlighting the therapeutic versatility attributed to certain macrofungi.

Historically, the indigenous peoples of Brazil were classified as non-mycophilic in the first ethnomycological studies [18, 69]. However, subsequent investigations demonstrated the consumption of mushrooms by several ethnic groups, especially among the Yanomami, in which species with high use values were recorded [12, 13, 18, 40]. In contrast, Andean populations of South America are widely recognized as mycophilic [70, 71].

The consumption of macrofungi has also been documented in different regions of Latin America, including the Colombian Amazon [66], the west coast of Chile [67], Venezuela [33], Mexico [43, 68, 72], and Ecuador [73]. Among the Yanomami, *Favolus brasiliensis* was identified as one of the most collected and consumed species and was later analyzed for its nutritional and food potential [27, 74].

### Return to the community: *Research does not end in the forest*

As the study progressed, reports and observations indicated that the Guajajara people made limited use of macrofungi and were often unaware of their potential uses. In response, the authors decided to share information about the importance and the various uses of macrofungi with the community, particularly in the school environment, where knowledge can be effectively disseminated.

**Fig. 5:**
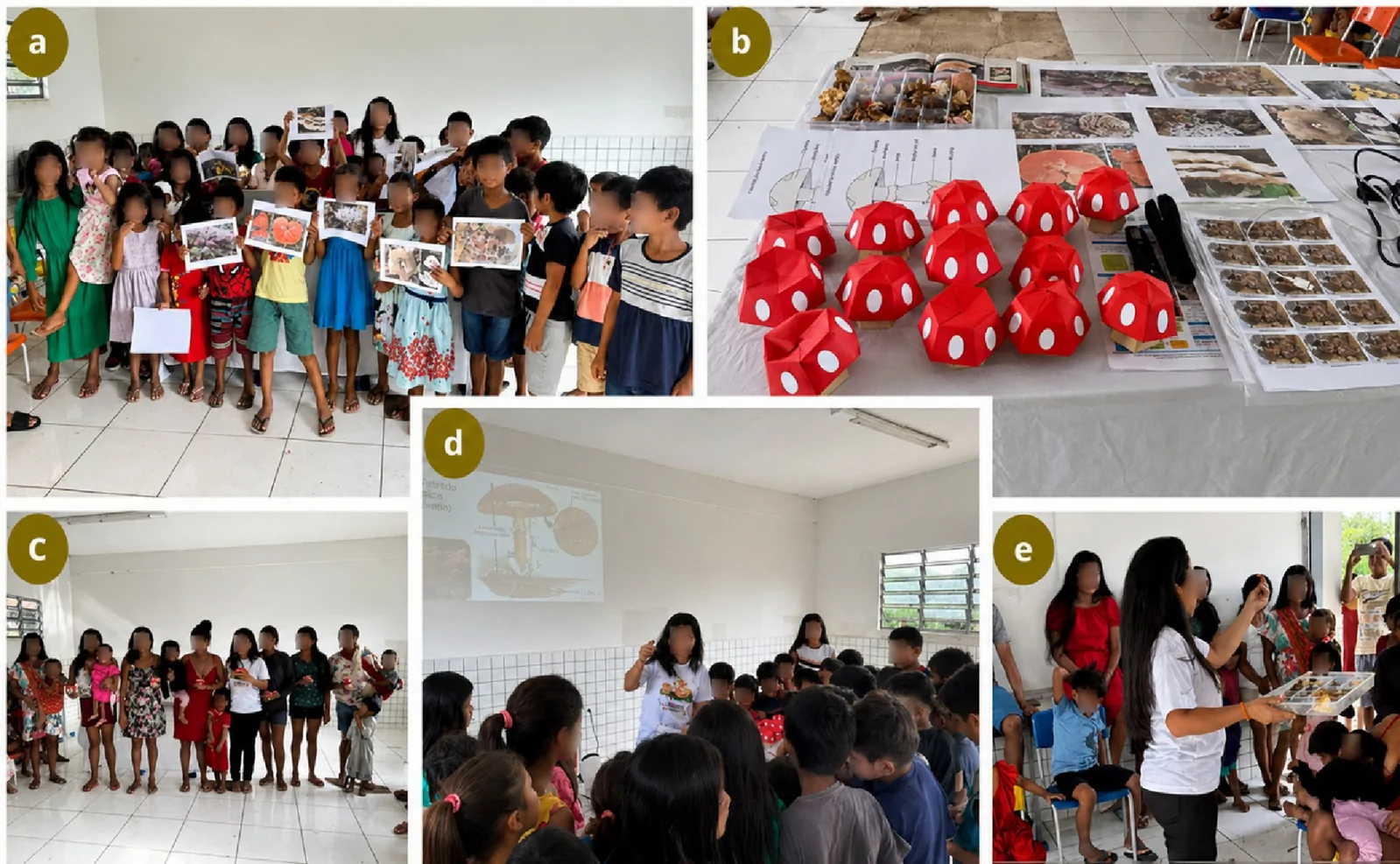
Workshop given at the Três Irmãos Indigenous Village School, of the Guajajara indigenous peoples of Barra do Corda, MA, Cana Brava Reserve. (**a**,** c)** official photo with the participants (children, young people and adults). (**b)** reinforcing visual resources, souvenirs and media. **(d**,** e)** researcher explaining the use of macrofungi with samples of the species.

At the end of the activity, the participants received souvenirs in the form of mushroom boxes as a symbolic memory of a unique moment: the return of the research to the community. Returning the research to the indigenous community represents a gesture of respect, ethics, and appreciation for traditional knowledge and for the fundamental role these communities play in biodiversity preservation (Fig. 5).

## Conclusions

The analyses carried out in this study revealed that the Guajajara indigenous people possess limited recognition of macrofungi in the environment in which they live, demonstrating a weak perception regarding the use of the various *urupés* found in the studied villages. Although macrofungi are no longer currently used as part of their diet, the Guajajara recognized that some species may have food potential, such as reports concerning the species *A. tremellosa*, which was mentioned as part of the diet of their ancestors.

The Guajajara indigenous people also use fungi for recreational activities, with mentions of *C. rugosa*, *G. harriotii*, and *G. javanicum*, and occasionally for medicinal purposes (*P. sanguineus* and *L. crinitus*), reporting greater use in the past, with applications related to the female reproductive system, kidneys, gastrointestinal tract, and skeletal system. The vertical mode of knowledge transmission is responsible for transferring information regarding the uses of macrofungi.

Based on the data obtained in this study, the Guajajara indigenous group does not demonstrate total aversion to macrofungi and therefore may be considered partially mycophilic (or non-mycophobic). However, the results also suggest that ethnomycological knowledge may be undergoing a process of erosion. The information documented here, including species recognition and reported uses, demonstrates the current state of this knowledge within the studied communities. Thus, this study contributes to the appreciation and conservation of local traditional knowledge of the indigenous peoples of Brazil regarding biodiversity and mycodiversity.

In the past, macrofungi were more frequently used by the Guajajara, especially as medicine. The use of pharmaceutical drugs and their easy accessibility may have replaced the traditional practice of using macrofungi among the indigenous people.

## Data Availability

All interview records, informed consent documents, and legal permits related to the interviews and the collection of fungi are archived at the Graziela Barroso Herbarium (TEPB) of the Federal University of Piauí, Brazil. The data produced or analyzed during this research can be made available upon request to the first author.
